# Regulation of ABA-Non-Activated SNF1-Related Protein Kinase 2 Signaling Pathways by Phosphatidic Acid

**DOI:** 10.3390/ijms21144984

**Published:** 2020-07-15

**Authors:** Maria Klimecka, Maria Bucholc, Justyna Maszkowska, Ewa Krzywińska, Grażyna Goch, Małgorzata Lichocka, Jadwiga Szczegielniak, Grażyna Dobrowolska

**Affiliations:** 1 Institute of Biochemistry and Biophysics, Polish Academy of Sciences, Pawińskiego 5a, 02-106 Warsaw, Poland; maria.klimecka@gmail.com (M.K.); maria.bucholc@gmail.com (M.B.); j.maszkowska@ibb.waw.pl (J.M.); ewakrzywa@gmail.com (E.K.); grazynagoch@gmail.com (G.G.); mlichocka@ibb.waw.pl (M.L.); jaga@ibb.waw.pl (J.S.); 2 Structural Biology Group, Biological and Chemical Research Centre, Department of Chemistry, University of Warsaw, Żwirki i Wigury 101, 02-089 Warsaw, Poland

**Keywords:** *Arabidopsis thaliana*, SnRK2-interacting calcium sensor, SCS, dehydrins, ERD14, PP2CA, SnRK2, SNF1-related protein kinases, phosphatidic acid, PA

## Abstract

Phosphatidic acid (PA) is involved in the regulation of plant growth and development, as well as responses to various environmental stimuli. Several PA targets in plant cells were identified, including two SNF1-related protein kinases 2 (SnRK2s), SnRK2.10 and SnRK2.4, which are not activated by abscisic acid (ABA). Here, we investigated the effects of PA on various elements of ABA-non-activated SnRK2 signaling. PA 16:0/18:1 was found to modulate the SnRK2 structure and the phosphorylation of some SnRK2 targets. Conversely, phosphorylation by the ABA-non-activated SnRK2s, of one of such targets, dehydrin Early Responsive to Dehydration 14 (ERD14), affects its interaction with PA and subcellular localization. Moreover, PA 16:0/18:1 modulates the activity and/or localization of negative regulators of the ABA-non-activated SnRK2s, not only of the ABA insensitive 1 (ABI1) phosphatase, which was identified earlier, but also of another protein phosphatase 2C, PP2CA. The activity of both phosphatases was inhibited by about 50% in the presence of 50 μM PA. PA 16:0/18:1 also impacts the phosphorylation and subcellular localization of SnRK2-interacting calcium sensor, known to inhibit SnRK2 activity in a calcium-dependent manner. Thus, PA was found to regulate ABA-non-activated SnRK2 signaling at several levels: the activity, phosphorylation status and/or localization of SnRK2 cellular partners.

## 1. Introduction

Phospholipids are not only the major components of cell membranes, but they also act as second messengers involved in plant responses to a variety of stimuli, including biotic and abiotic stresses. Among them is phosphatidic acid (PA), which attracted increasing attention in recent years as a regulator of signal transduction, membrane trafficking, secretion, and cytoskeletal rearrangements (for reviews, see References [1,2,3,4,5,6,7,8,9,10,11,12,13]). It is well documented that PA accumulates rapidly (within seconds to minutes) in plant cells in response to environmental stresses (e.g., salinity, drought, wounding, cold, freezing, and pathogen attack). In plants, PA can be generated via several distinct routes: via phospholipases D (PLD) which hydrolyze structural lipids to PA and a free head group, via a sequential action of phospholipases C (PLC) and diacylglycerol kinase (DAGK), and also via de novo synthesis by lysophosphatidyl acyltransferases from the glycerol-3-phosphate (Gro3P) pathway-derived lysophosphatidic acid (LPA) (for reviews, see References [6,13,14]). Activation of these pathways determines the timing, localization, and accumulation of specific molecular species of PA. The accumulation of PA in response to stress is transient; it is controlled not only by the rate of synthesis but also by its metabolism. The level of PA can be downregulated via hydrolysis by PA hydrolases, dephosphorylation by lipid phosphate phosphatases, phosphorylation by PA kinase, and degradation by PLA_2_ (for a review, see Reference [6]). PA was shown to transmit signals by changing the localization of the target protein (in most cases, by recruiting it to membranes), by altering protein conformation, which can, e.g., modulate its enzymatic activity, and by affecting the curvature of membranes [6].

Numerous reports showed an involvement of PA in the response to salinity and drought [15,16,17,18,19,20,21] (for reviews, see References [1,6,22,23,24,25]). Several proteins playing an important role in abscisic acid (ABA) responses are PA targets. Studies on ABA signaling in *Arabidopsis thaliana* revealed that PA interacts with and affects the activity and localization of a key negative regulator of the ABA signaling in *Arabidopsis thaliana*—the PP2C phosphatase ABI1 (ABA insensitive 1) [15]. The binding of PA inhibits the enzymatic activity of ABI1 and targets it to the plasma membrane in response to ABA. Moreover, PA binds to and enhances the activity of an Nicotinamide Adenine Dinucleotide Phosphate (NADPH) oxidase, Respiratory burst oxidase homolog protein D (AtRbohD), thereby increasing the level of reactive oxygen species (ROS) and, as a consequence, causing stomatal closing in response to ABA [26]. Additionally, a crosstalk between phospholipase D (PLD) and sphingosine kinase (SPHK) was shown. SPHK is responsible for phosphorylation of sphingolipids and, consequently, the synthesis of long-chain-1-phosphates such as phytosphingosine-1-phosphate (phyto-S1P) which, similarly to PA, is involved in the plant response to ABA. Guo et al. [27] showed that PA, a product of PLD, interacts with SPHK and stimulates its activity, whereas SPHK and phyto-S1P act upstream of PLDα1 in stomatal movement in response to ABA [27,28]. These results indicate that PLDα and SPHK, as well as their products, amplify the ABA signal for stomatal closing via a positive feedback loop. Moreover, it was shown that ABA-induced microtubule depolymerization and stomatal closing are impaired in a *pldα1* mutant [29]. All these findings indicate that, by regulating various elements of the ABA signaling network, PA acts as a positive regulator of stomatal closing in plants exposed to drought or other stresses inducing ABA accumulation.

In response to salt stress, PA produced by PLDα1 stimulates the activity of *Arabidopsis* mitogen-activated protein kinase 6 (MPK6) which in turn phosphorylates Salt Overly Sensitive 1 (SOS1) [17], an Na^+^/H^+^ antiporter responsible for Na^+^/K^+^ homeostasis. Additionally, upon salt stress, PA binds to MPK6 upstream kinases MKK7 and MKK9, enhances their kinase activity, and induces translocation to the plasma membrane [30]. PA is also essential for proper microtubule organization by interacting with microtubule-associated protein MAP65-1 [20], a substrate of several different protein kinases (for a review, see Reference [31]), including MPK6, which itself is a PA target, as mentioned above. 

It was shown that primary roots of single *pldα1* or *pldδ* and double *pldα1*/*pldδ* knockout *Arabidopsis* mutant plants growing in saline conditions are significantly shorter than those in the wild type (wt) [32]. Similarly, *pldα3* mutant plants are more susceptible to salt stress than the wt; in this mutant, both the primary root growth and the number of lateral roots are more reduced when exposed to salt stress than in wt plants [33]. Similar phenotypes were observed for *snrk2.4* and *snrk2.10* mutants (impaired in Sucrose non-fermenting 1 (SNF1)-related protein kinase 2.4 (SnRK2.4) and SnRK2.10, respectively) grown in salt stress conditions [19]. The primary roots of the *snrk2.4* seedlings were shorter, whereas the number of lateral roots of *snrk2.10* was lower than in the wt Col-0 seedlings grown in medium supplemented with 115 mM NaCl. SnRK2.4 and SnRK2.10 were identified as PA targets [34], and the results presented by Mclaughlin et al. [19] and Julkowska et al. [21] indicate interplay between these kinases and PA in the regulation of root architecture in response to salt stress. Another protein interacting with PA in response to salt stress in *Arabidopsis* roots is glyceraldehyde-3-phosphate dehydrogenase (GAPDH) [7]. Kim et al. [35] showed that PA affects primary root growth partially by inducing proteolytic degradation of GAPHD. At this point, it should be mentioned that, in tobacco, GAPDH is one of the cellular partners of *Nicotiana tabacum* osmotic stress-activated protein kinases (NtOSAK) [36], a close homolog of *Arabidopsis* SnRK2.4 and SnRK2.10 kinases. Recently, we identified several potential substrates of SnRK2.10 in plant roots exposed to NaCl, including dehydrins Early Responsive to Dehydration 10 (ERD10) and ERD14 [37]. The dehydrins also bind PA, which directs them to cellular membranes in order to protect the cell against the negative effects of stress [38,39,40]. 

SnRK2s are considered to be major regulators of the plant responses to various abiotic stresses (e.g., drought, salinity, stress induced by heavy metals) and of ABA-dependent plant development [19,41,42,43,44,45] (for reviews, see References [46,47,48,49,50]). In *Arabidopsis* and in rice, there are 10 members of the SnRK2 family. Based on a phylogenetic analysis, they are divided into three groups. This classification correlates with their response to ABA; members of group 1 are not activated by ABA (SnRK2.4 and SnRK10, as mentioned before, belong here), those of group 2 are not activated or activated very weakly by ABA (depending on plant species), and those of group 3 are strongly activated by ABA. The most extensively studied are members of group 3 (SnRK2.2/SRK2D, SnRK2.3/SRK2I, and SnRK2.6/SRK2E in *Arabidopsis*) [41,42,43]. It was shown that the ABA-dependent SnRK2s control the response to water deficit mainly by regulating of stomatal closing via phosphorylation of anion and cation channels [Slow Anion Channel-Associated 1 (SLAC1), Quick anion channel 1 (QUAC1), cation K^+^ channel 1(KAT1)], as well as a vacuolar antiporter of the chloride channel (CLC) AtCLCa [51,52,53,54,55], and via the regulation of the expression of ABA- and water stress-induced genes by phosphorylation of ABA-responsive transcription factors (for reviews, see References [46,48,49,56]. The major role in the regulation of the ABA-dependent stomatal closing is played by SRK2E/SnRK2.6/ Open Stomata 1 (OST1). The kinase not only regulates the activity of substrates mentioned above but also induces the production of ROS [57] (for a review, see Reference [58]), which are indispensable in this process. There are strong indications that the NADPH oxidase AtRbohF is a SnRK2.6/OST1 substrate [59,60]. Therefore, it is highly likely that SnRK2 together with other kinases phosphorylating AtRbohs, such as Calcineurin B-Like Protein (CBL)-Interacting Protein Kinases (CIPKs) [60,61] and Calcium-Dependent Protein Kinases (CDPKs) [62], in concert with PA [26], regulates the oxidase activity in response to drought and salinity. It was also shown that seed maturation and germination are controlled by both PA [63,64,65] and the group 3 SnRK2s [41,43,66]. Several negative regulators of SnRK2s are known, among them phosphoprotein phosphatases of the PP2C [67,68,69,70] and okadaic acid-sensitive phosphatase(s) from the phosphoprotein phosphatase PPP families [69,71], and SnRK2-interacting calcium sensor (SCS) [72,73]. As already mentioned, the PP2C phosphatase ABI1 is regulated by PA [15]. Moreover, subunits of PP2A, which belong to the PPP family, were shown to interact with PA [34,74].

All these data indicate that PA plays an important role in the regulation of SnRK2s pathways activated in response to drought and salinity, both in an ABA-dependent and an ABA-independent manner. Here, to gain more information on the participation of PA in the regulation of the SnRK2 pathways, we studied the binding of various PA species to several members of the SnRK2 family and the impact on their activity. We focused on the ABA-non-activated SnRK2s from group 1, which were earlier found to be PA targets, as well as on SnRK2.8 from group 2, not yet studied with respect to PA binding. We analyzed the effects of PA on the SnRK2s themselves, as well as on the phosphorylation and localization of their partners. Our data revealed that PA 16:0/18:1 is involved in the regulation of ABA-non-activated SnRK2 pathways at various levels by modulating the activity and/or localization of diverse SnRK2 regulators and the phosphorylation and subcellular localization of SnRK2 substrates.

## 2. Results

### 2.1. ABA-Non-Activated SnRK2s, Their Substrates ERD10 and ERD14, and PP2C Clade A Phosphatases ABI1 and PP2CA Preferentially Interact with PA 16:0/18:1

**Figure 1:**
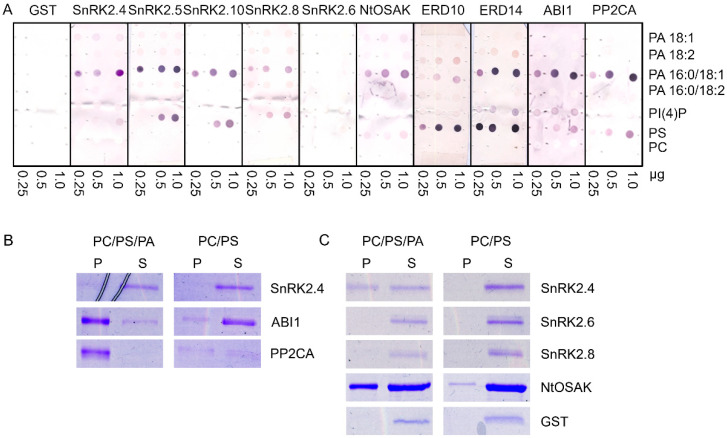
Binding of selected proteins engaged in SNF1-related protein kinase 2 (SnRK2) signaling to phospholipids. The proteins studied were group 1 SnRK2s (*Arabidopsis* SnRK2.4, SnRK2.5, SnRK2.10, and tobacco NtOSAK), *Arabidopsis* SnRK2.8 (group 2), *Arabidopsis* SnRK2.6 (group 3), the regulatory phosphatases abscisic acid (ABA) insensitive 1 (ABI1) and protein phosphatase 2C A (PP2CA), as well as SnRK2s substrates, the dehydrins Early Responsive to Dehydration 10 (ERD10) and ERD14. The proteins in fusion with glutathione S-transferase (GST) were bacterially expressed and purified. (**A**) Phospholipid blot assay. Phospholipids (0.25, 0.5, 1.0 µg) were spotted onto nitrocellulose and incubated with 10 µg/mL of GST fusion proteins. Bound proteins were detected by immunoblotting using anti-GST antibodies. GST was used as a negative control. (**B**,**C**) Liposome assay. Liposomes composed of phosphatidylcholine (PC)/phosphatidylserine (PS)/phosphatidic acid (PA) 16:0/18:1 (4:1:4.4) or PC/PS (4:1) were incubated with 1.3 μg of indicated proteins and then pelleted by centrifugation. Proteins in the pellet (P) and supernatant (S) were separated by SDS-PAGE and stained with Coomassie Brilliant Blue. In (**C**), ten-fold more liposomes was used per assay than in (**B**). Data represent one of at least three independent experiments showing similar results.

It was established that two *Arabidopsis thaliana* kinases, SnRK2.4 and SnRK2.10, which belong to group 1 of the SnRK2 family (ABA-non activated kinases), interact with PA, whereas the ABA-activated kinase SnRK2.6 does not [19,34], suggesting that this interaction is specific for the SnRK2s that are not activated in response to ABA [21]. However, it was indicated that various molecular species of PA differ in their affinity toward proteins [15,75]. Therefore, we tested the binding of four biologically relevant PA species (with different fatty acid chains) to various members of the *Arabidopsis* SnRK2 family: SnRK2.4, SnRK2.5, and SnRK2.10 (from group 1), SnRK2.8 (from group 2), SnRK2.6 (from group 3), and additionally NtOSAK, a tobacco SnRK2 closely related to SnRK2.4/SnRK2.10. We analyzed the binding of PA 18:1, 18:2, 16:0/18:1, and 16:0/18:2. We also analyzed two other acidic phospholipids: phosphatidylinositol 4-phosphate (PI(4)P) and phosphatidylserine (PS), with the zwitterionic phosphatidylcholine (PC) as a negative control. Additionally, we tested the interaction of the phospholipids with two phosphatases from clade A of the *Arabidopsis* PP2C family, which are established to be negative regulators of the ABA-activated [67,68] and ABA-non-activated [69,70] SnRK2s, ABI1 and PP2CA. Notably, ABI1 is a known PA target [15]. Two substrates of ABA-non-activated SnRK2s identified by Reference [37], the dehydrins ERD10 and ERD14, were also tested. It should be mentioned that the interaction between phospholipids (PA and PS) and the dehydrins studied here was established before [38,39,40,76] but without distinguishing PA species. All the proteins to be studied were produced in *Escherichia coli* in fusion with glutathione S-transferase (GST) to facilitate their quantification; therefore, GST was used as a negative control in the binding assays. The phospholipid blot assays revealed that all the proteins studied with the exception of SnRK2.6 preferentially bound to PA 16:0/18:1; no significant binding to other PA species tested was observed (Figure 1A). The binding of SnRK2.8 was weaker than that of the other proteins. None of the proteins studied bound to PC, which was used as a negative control, but we observed some interaction of the ABA-non-activated SnRK2s and SnRK2.8, as well as ABI1 and PP2CA, with PS. In agreement with earlier data [40,77], the two dehydrins bound both PA and PS. In our hands, ERD10 preferably bound PS, while ERD14 preferably bound PS and PA 16:0/18:1. SnRK2.6 did not bind to any lipid. As expected, GST did not either. In the case of ABI1, we observed a weak signal indicating an interaction with PI(4)P; however, a lack of a correlation between the amount of PI(4)P spotted and the signal intensity makes interpretation of this result difficult.

The interactions between some of the proteins studied and PA 16:0/18:1 were verified by a liposome assay. For the assay, we chose *Arabidopsis* SnRK2s, one from each group (SnRK2.4 from group 1, SnRK2.8 from group 2, and SnRK2.6 from group 3), NtOSAK, tobacco SnRK2 from group 1 NtOSAK, the phosphatases (PP2CA and ABI1), and GST as a negative control. In previous PA–protein binding studies, liposomes containing 640 nmol [78], 400 nmol, and, in some experiments, ten-fold less (40 nmol) [21] lipids (PC, PS, and PA) per sample were used. We used the liposomes produced from PC and PS mixed with PA 16:0/18:1, with 64 nmol (Figure 1B and Figure S1, Supplementary Materials) or 640 nmol (Figure 1C) of total lipids per sample. Liposomes without PA were used as a control. After incubation with the selected proteins, the liposomes were pelleted and the proteins in the pellet and supernatant were identified. The results were basically in agreement with the blot assay results; the ABA-non-activated SnRK2s (SnRK2.4 and NtOSAK) and the phosphatases (ABI1 and PP2CA) did bind to the PA-containing liposomes, whereas SnRK2.6 and GST did not (Figure 1B,C). The binding was not consistent for SnRK2.8. We observed its weak binding to PA 16:0/18:1 using the blot assay and very weak or no binding when PA was incorporated into the liposomes (Figure 1C and Figure S1, Supplementary Materials). This suggests that the PA–SnRK2.8 binding is very weak at best. It should be noted that both phosphatases (ABI1 and PP2CA) bound to the liposomes containing 64 nM of lipids, while, in the case of SnRK2.4 and NtOSAK, an evident interaction was observed only with a higher amount of lipids, indicating a higher affinity of the phosphatases compared with the kinases for PA (Figure 1B,C and Figure S1, Supplementary Materials). It should be mentioned that we also observed some binding of PP2CA to the control PC/PS liposomes, in agreement with the lipid overlay assay where PP2CA bound weakly to PS. Similar findings were reported previously by Zhang et al. [15], who studied the binding between PA and ABI1. 

In all further experiments, we exclusively used the 16:0/18:1 species of PA, unless indicated otherwise.

### 2.2. PA Binding Modulates SnRK2.4 Structure

**Figure 2:**
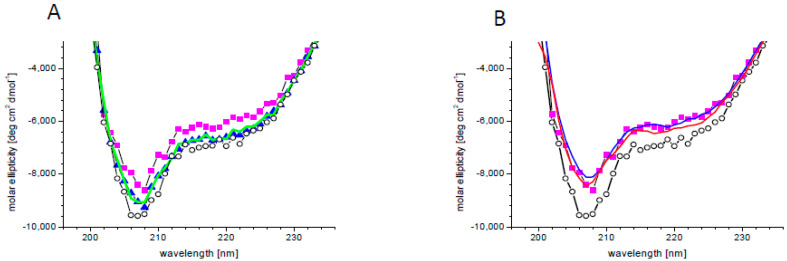
Changes in SnRK2.4 conformation upon PA binding. (**A**) Circular dichroism (CD) spectra of SnRK2.4 kinase without lipids (open circles) and with 5 μM lipid solution of PA 16:0/18:1 (magenta squares), PA 18:1 (blue triangles), and PC (green line). (**B**) CD spectra of SnRK2.4 kinase without lipids (open circles) and in the presence of PA 16:0/18:1 at 5 (magenta squares), 10 (blue line), and 50 (red line) μM.

There are indications that PA binding influences the activity or localization of its specific targets [21,30,78,79,80,81,82]. A change in protein activity requires a change in its structure, which may be subtle or substantial. In order to check the effect of PA on the SnRK2.4 structure, circular dichroism (CD) spectra were determined without or with PA 18:1/16:0 and additionally with PA18:1 which, according to the phospholipid blot assay, does not bind or binds very weakly to SnRK2.4, as well as PC as a control. The secondary structure content of SnRK2.4 determined from its CD spectrum (Figure 2) using the CDNN program was as follows: α-helix 29%, β-sheet 19%, β-turn 18%, and random coil 34%. In the presence of 5 μM PA 16:0/18:1, the whole spectrum moved upward by ca. 10%, indicating modest structural changes (Figure 2A). Notably, the ellipticity at 222 nm, related to α–helical fragments, was decreased somewhat more (by ca. 15%), indicating a substantial decrease of the α–helical content in SnRK2.4 upon interaction with PA 16:0/18:1. As expected, the other lipids tested had a negligible effect on the CD spectra, which confirmed the specificity of the SnRK2.4 interaction with PA 16:0:18:1. When higher doses of PA 16:0/18:1 (10 and 50 μM) were used, no additional changes in the spectra were observed (Figure 2B), indicating that the lowest concentration used (5 μM) was saturating. 

### 2.3. PA Affects Phosphorylation of SnRK2 Targets

PA was shown to modulate the activity of several protein kinases involved in signal transduction in plants [17,30,78,79,80]. Since we found that PA affected the structure of SnRK2.4, and the accumulation of PA in plant cells in response to salinity was reported to correlate with the activation of SnRK2.4/10 in *Arabidopsis* [19], as well as with the activation of NtOSAK in tobacco cells, [83], we reasoned that PA could have an effect on the activity of SnRK2s. 

**Figure 3:**
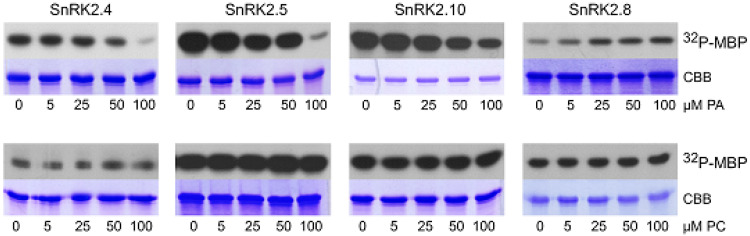
Effect of PA on activity of SnRK2s. The in vitro activity of bacterially expressed and purified kinases in fusion with GST (ABA-non-activated SnRK2.4, SnRK2.5, and SnRK2.10, and ABA weakly activated SnRK2.8, about 1 μg each) was measured in the presence of increasing amounts of PA 16:0/18:1 (upper panel) or PC (lower panel) in a reaction mixture with myelin basic protein (MBP) and [γ^32^P]ATP as substrates. Reaction products were separated by SDS-PAGE, and the extent of MBP phosphorylation was determined by autoradiography. ^32^P-MBP—autoradiography; CBB—Coomassie Brilliant Blue staining. Data represent one of three independent experiments showing similar results.

We analyzed the effect of PA, as well as PC as a control, on the phosphorylation of myelin basic protein (MBP), a substrate commonly used for determination of kinase activity, using three ABA-non-activated SnRK2s (SnRK2.4, SnRK2.5, and SnRK2.10), as well as SnRK2.8. PA inhibited the MBP phosphorylation using all ABA-non-activated SnRK2s studied; however, the effect was significant only when PA concentration was relatively high (Figure 3). In contrast, the activity of SnRK2.8 was slightly enhanced by PA. No significant effect of PC on the MBP phosphorylation by any of the kinases tested was visible. Even though, in the case of MBP phosphorylation, the effect of PA was significant at rather high concentrations of the phospholipid, we considered that it could be more pronounced for physiological SnRK2 targets. 

Therefore next we studied the effect of PA on the phosphorylation of bona fide cellular SnRK2 targets. We chose two dehydrins (ERD10 and ERD14) shown recently to be phosphorylated by ABA-non-activated SnRK2s in *Arabidopsis* roots in response to salinity [37], as well as SnRK2-interacting calcium sensor-A (AtSCS-A), a negative regulator of SnRK2 activity whose phosphorylation by SnRK2s was identified by Bucholc et al. [72]. 

**Figure 4:**
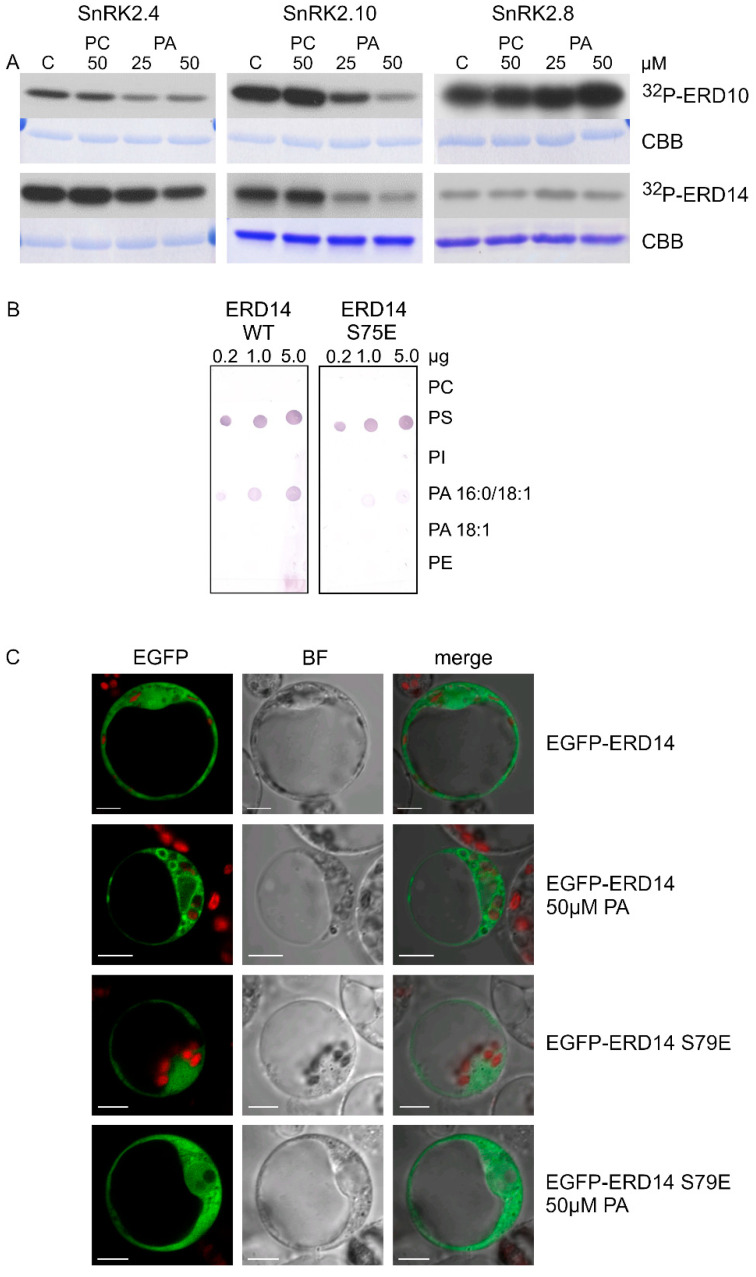
Mutual relationships between PA and phosphorylation and subcellular localization of dehydrins. (**A**) Effect of PA on the phosphorylation of ERD10 and ERD14 by various SnRK2s. Phosphorylation of the dehydrins by the ABA-non-activated SnRK2s SnRK2.4 and SnRK2.10 and the ABA weakly activated SnRK2 SnRK2.8 was analyzed in vitro in the absence or presence of 25 and 50 μM PA 16:0/18:1 or PC in the reaction mixture. The kinases and dehydrins were produced in *Escherichia coli*, and purified proteins in fusion with GST were used. Reaction products were separated by SDS-PAGE, and phosphorylation was determined by autoradiography. ^32^P-ERD10/ERD14—autoradiography; CBB—Coomassie Brilliant Blue staining. Data represent one of three independent experiments showing similar results. (**B**) Phospholipid binding by ERD14 and ERD14 S79E. Binding of different phospholipids by ERD14 and ERD14 S79E was analyzed using a lipid overlay assay. Nitrocellulose membranes with increasing doses of immobilized phospholipids, as indicated, were incubated with GST-fused dehydrins, and their binding was determined. Next, proteins bound to phospholipids were determined using anti-GST antibodies. Data represent one of two independent experiments showing similar results. (**C**) Effect of PA on subcellular localization of ERD14 and ERD14 S79E. Protoplasts isolated from T87 *Arabidopsis* cells transiently transformed expressing EGFP-ERD14 or EGFP-ERD14 S79E were treated or not with PA 16:0/18:1 and analyzed by confocal microscopy. BF—bright field; bar = 10 µm. Data represent one of three independent experiments showing similar results.

In vitro phosphorylation of ERD10 and ERD14 catalyzed by SnRK2.4, SnRK2.10, and SnRK2.8 was analyzed in the absence or presence of PA, with PC used as a control. PA significantly inhibited the phosphorylation of both dehydrins by SnRK2.4 and SnRK2.10, but not by SnRK2.8 (Figure 4A). Notably, the inhibition was clearly visible already at 25 μM PA, confirming our expectations that the effect of PA on the SnRK2 phosphorylation could be more pronounced for natural substrates of the kinases. As expected, PC had no effect on the phosphorylation.

**Figure 5:**
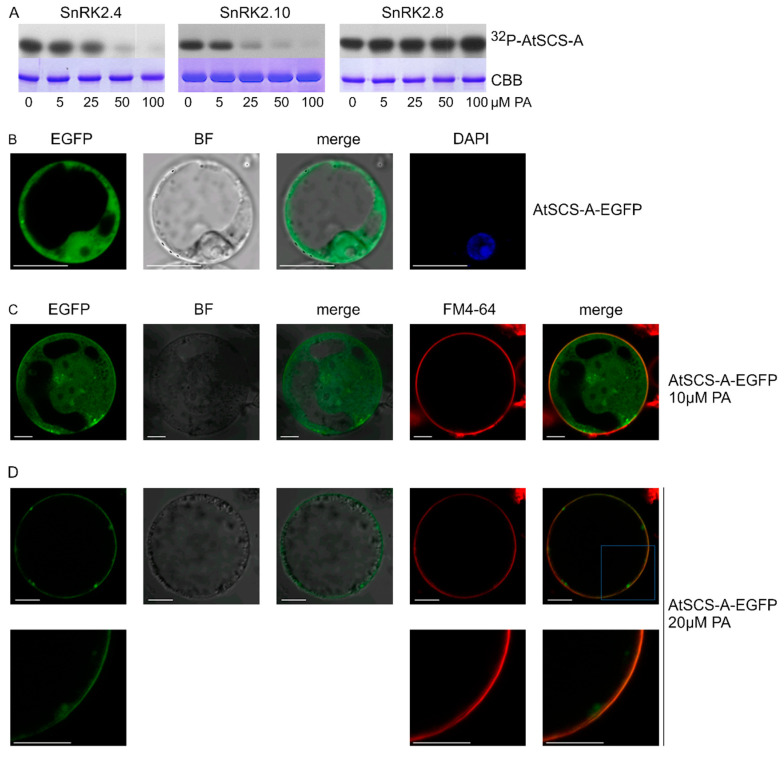
Effect of PA on phosphorylation of SnRK2-interacting calcium sensor-A (AtSCS-A) by ABA-non-activated SnRK2s and its subcellular localization. (**A**) Phosphorylation of AtSCS-A by recombinant GST-fused kinases SnRK2.4, SnRK2.10, and SnRK2.8 was analyzed in vitro in the presence of increasing concentrations of PA 16:0/18:1 in the reaction mixture using AtSCS-A and [γ^32^P]ATP as substrates. Reaction products were separated by SDS-PAGE, and AtSCS-A phosphorylation was determined by autoradiography. ^32^P-AtSCS-A—autoradiography; CBB—Coomassie Brilliant Blue staining. Data represent one of three independent experiments showing similar results. (**B**–**D**) Subcellular localization of AtSCS-A in the absence (**B**) or presence (**C**,**D**) of PA. Protoplasts isolated from T87 *Arabidopsis* cells transiently expressing AtSCS-A-EGFP were treated with PA 16:0/18:1 at 10 μM or 20 μM and analyzed by confocal microscopy. In C and D, the membrane was stained with FM4-64. BF—bright field; bar = 10 µm. Data represent one of three independent experiments showing similar results.

As mentioned, another SnRK2 substrate studied with respect to the effect of PA on its phosphorylation was AtSCS-A. In vitro phosphorylation of AtSCS-A obtained in a bacterial system by the ABA-non-activated SnRK2s, SnRK2.4 and SnRK2.10, as well as by SnRK2.8 from group 2 of the SnRK2 family, was analyzed in the absence or presence of PA, with PC used as a control. As in the case of dehydrins, PC did not have a significant effect on the AtSCS-A phosphorylation (Figure S2, Supplementary Materials), while PA significantly inhibited the phosphorylation by SnRK2.4 or SnRK2.10, but again not by SnRK2.8 (Figure 5A).

### 2.4. Interplay between PA Binding and Phosphorylation of Dehydrins

As already mentioned, it is well established that dehydrins bind phospholipids [38,39,40]. Additionally, it was shown that this interaction can be modulated by phosphorylation [40]. Previously, we showed that SnRK2.4 and SnRK2.10 preferably phosphorylate S106 in ERD10 and S79 in ERD14 [37]. Our data suggested that the phosphorylation of ERD14 at S79 upon salt stress could affect its interaction with membranes and modulate the salt-induced membrane remodeling [37]. An analysis of the subcellular localization of EGFP-ERD14 transiently expressed in *Nicotiana benthamiana* or *Arabidopsis* protoplasts showed that, in response to salt stress, a fraction of ERD14 relocates to nuclei and some associates with the membrane of large bulb-like vesicles [37]. A mutated form of ERD14 with a phosphomimetic substitution (ERD14 S79E) also showed a cytoplasmic/nuclear localization, while the membrane association was much less visible [37]. Those results suggest that phosphorylation of ERD14 at S79 has a negative effect on the membrane binding. ERD10 showed a different behavior, as it was nearly exclusively cytoplasmic even after salt stress, and the phosphomimetic substitution S106E did not change this localization [37]. We remind here that the two dehydrins were found to exhibit different preferences for phospholipid binding: ERD14 for PA 16:0/18:1 and ERD10 for PS (Figure 1).

The behavior of the S79E variant of ERD14 prompted us to analyze the effect of ERD14 phosphorylation at S79 on phospholipid binding. We performed the lipid blot assay for wild-type ERD14 and its phosphomimetic variant ERD14 S79E, both obtained in a bacterial system. The negatively charged residue in ERD14 S79E weakened the binding with PA 16:0/18:1 but not with PS (Figure 4B), indicating that phosphorylation of ERD14 at S79 likely disturbs the interaction with membranes in a PA-specific manner.

Additionally, we analyzed the subcellular localization of EGFP-ERD14 and EGPF-ERD14 S79E transiently expressed in *Arabidopsis* protoplasts exposed to 50 μM PA. The wild-type ERD14 was clearly visible in proximity to membranes of large vesicles, whereas ERD14 S79E only rarely showed such localization (Figure 4C). Taken together, the above results indicate that PA inhibits the phosphorylation of dehydrins via the ABA-non-activated SnRK2s and, in turn, the phosphorylation affects ERD14–PA binding.

### 2.5. PA Impacts Subcellular Localization of AtSCS-A

We analyzed the effect of PA on the subcellular localization of AtSCS-A. Previously, Bucholc et al. [72] showed that AtSCS-A localized to the cytoplasm and the nucleus. Here, we analyzed the subcellular localization of AtSCS-A-EGFP transiently expressed in *Arabidopsis* protoplasts before and after treatment with PA. The effect of PA on AtSCS-A localization was dramatic; in protoplasts not exposed to PA, AtSCS-A was distributed in the nucleus and the cytoplasm (Figure 5B), whereas after treatment with 20 μM PA, AtSCS-A-EGFP was no longer found in the nucleus and most of it assembled close to the plasma membrane (Figure 5C,D). We propose an indirect effect of PA on the AtSCS-A localization, since no direct binding between AtSCS-A and PA could be observed (Figure S3, Supplementary Materials). Instead, an unknown partner protein of AtSCS-A interacting with PA could be involved. 

### 2.6. PA Inhibits PP2CA and Modulates Its Subcellular Localization

It was established that SnRK2s are negatively regulated by clade A PP2C phosphatases both through a direct interaction and through dephosphorylation of specific residues in the kinase activation loop, whose phosphorylation is required for the SnRK2 activity [67,68]. Upon stress (e.g., drought, salinity), the level of ABA increases, while ABA receptors from the REGULATORY COMPONENTS OF ABA RECEPTOR / PYRABACTIN RESISTANCE 1 PYR) / PYR1-like RCAR/PYR1/PYL family form a complex with ABA and with clade A PP2C phosphatases, thus inducing a conformation change which causes the phosphatases inactivation allowing autophosphorylation and activation of the ABA-dependent SnRK2s [46,84,85,86,87,88].

**Figure 6:**
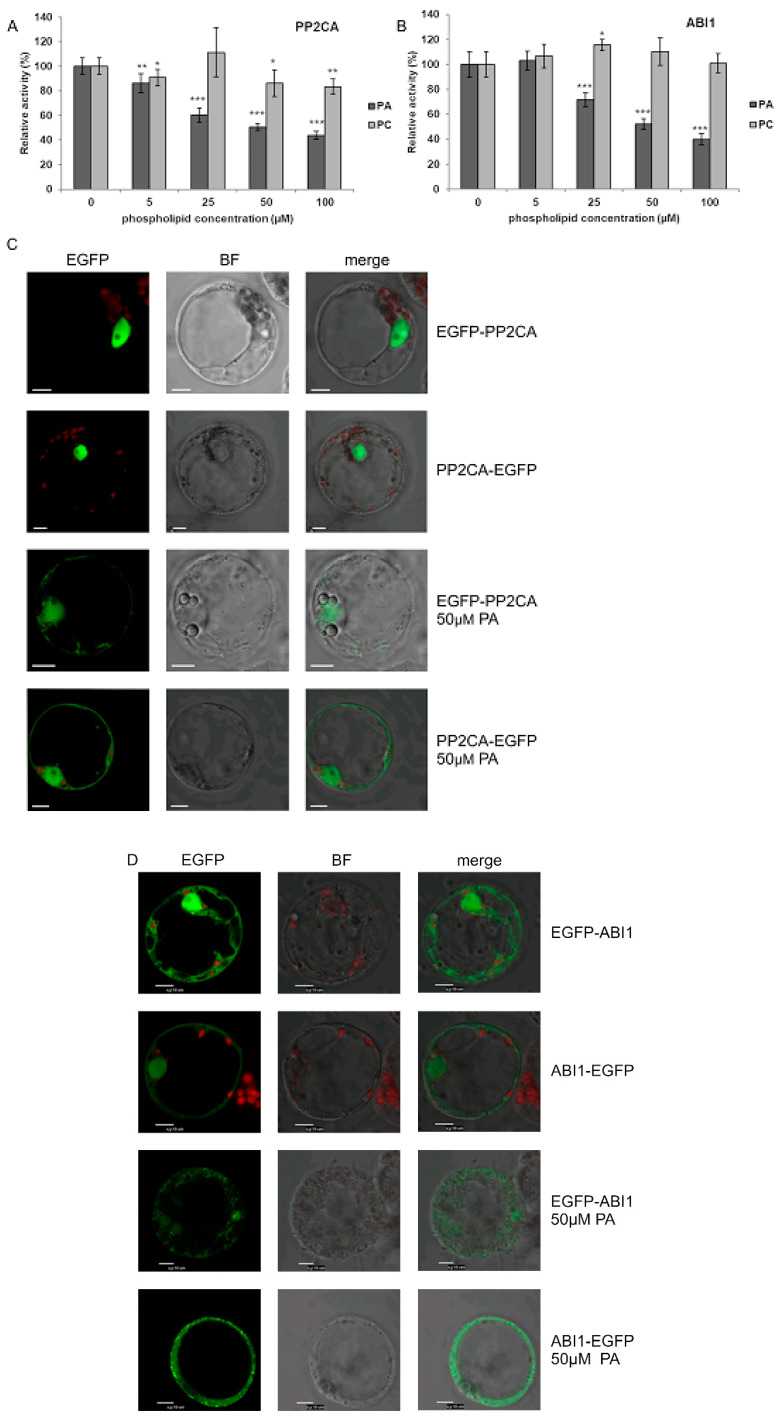
Effect of PA on the activity and subcellular localization of PP2CA phosphatase. (**A**,**B**) Effect of PA and PC on the activity of PP2CA (**A**) with ABI1 (**B**) used as a control. The activity of recombinant GST-fused phosphatases was determined with increasing doses of PA 16:0/18:1 or PC, using the ProFluor^®^ Ser/Thr PPase Assay. Average values ± standard error (SE) of three independent experiments are shown. For the statistical analysis, a Student *t*-test was applied. The asterisks indicate significant differences from control without lipids (* *p* < 0.05, ** *p* < 0.01, *** *p* < 0.001). (**C**,**D**) Effect of PA on subcellular localization of PP2CA (**C**) and ABI1 (**D**). Protoplasts isolated from T87 *Arabidopsis* cells were transiently transformed with plasmid encoding EGFP-PP2CA, PP2CA-EGFP, EGFP-ABI1, or ABI1-EGFP, and the localization of respective proteins after adding 50 μM PA 16:0/18:1 was analyzed by confocal microscopy. BF—bright field; bar = 10 µm. Data represent one of three independent experiments showing similar results.

Krzywińska et al. [69,70] revealed that two clade A PP2Cs, ABI1 and PP2CA, regulate the activity of the ABA-non-activated SnRK2s as well. However, the mechanism of regulation of these phosphatases in response to osmotic stress in an ABA-independent manner is still enigmatic. One of the mechanisms leading to inhibition of PP2Cs in an ABA-independent manner could involve their interaction with PA. Zhang et al. [15] showed that ABI1 interacts with PA and this interaction causes inhibition of the phosphatase. Our present results revealed that PA also interacts with PP2CA (Figure 1). Therefore, we analyzed the effect of PA (and PC as a control) on PP2CA activity and, as a positive control, on the activity of ABI1. The activity of recombinant GST-PP2CA and GST-ABI1 was determined in the presence of different concentrations of PA or PC. PC had no or a minimal effect on the activity of the phosphatases, while PA (50 µM) inhibited the activity of PP2CA (Figure 6A) and ABI1 (Figure 6B) by 50–60%. The extent of inhibition of both phosphatases by different doses of PA resembled the results for ABI1 presented by Zhang et al. [15]. 

In order to check if, similarly to ABI1, the interaction of PP2CA with PA influences its subcellular localization, we again employed the transient expression system in *Arabidopsis* T87 cell protoplasts. To avoid artefacts, two fusions with EGFP were investigated, EGFP-PP2CA and PP2CA-EGFP. Under control conditions both PP2CA constructs were nearly exclusively nuclear, whereas, following treatment with PA for 1 h, they were also found in the cytoplasm (Figure 6C). For ABI1 (Figure 6D), the results confirmed the earlier findings of Reference [15]. 

## 3. Discussion

Plants respond to challenging environmental conditions via activation of various defense pathways. In many of them, plant-specific kinases, SnRK2s, in concert with other kinases and diverse signaling molecules, are involved. There are ample data on the role and mechanisms of regulation of ABA-dependent SnRK2s in response to stress, especially water deficit, whereas much less is known regarding the SnRK2s not activated by ABA, members of group 1 of the SnRK2 family. Their physiological role was the subject of surprisingly few studies. It was reported that ABA-non-activated kinases are involved in the plant response to salinity [19,21,37,89,90,91], stress induced by cadmium ions [45], and osmotic stress [92]. The most striking feature of these kinases is their strong, extremely rapid, and transient activation in response to salinity; SnRK2.4 and SnRK2.10 are fully active in *Arabidopsis* roots already after one minute of treatment with NaCl [19]. Similarly, NtOSAK, a tobacco close relative of SnRK2.4/10, is activated within about two minutes after NaCl addition to BY-2 cells in suspension [83]. These results indicate that the ABA-non-activated SnRK2s likely have an important role in early response to salinity and that their activity is strictly controlled inside the cell. Several protein phosphatases, mostly from clade A of the PP2C family, were identified as negative regulators of the ABA-dependent SnRK2s [67,68,71]. Some of them, namely, ABI1 and PP2CA, were found to inhibit also the activity of the ABA-non-activated kinase SnRK2.4 [69,70]. Therefore, activation of SnRK2s requires inhibition of these phosphatases. The mechanism of this inhibition in response to ABA is well understood [46,86], but how it can occur in an ABA-independent manner remains to be determined. Previously published data indicated that ABA-non-activated SnRK2s and the phosphatase ABI1 inhibiting their activity bind PA [15,21]. Therefore, we reasoned that PA could act as a regulator of the ABA-independent SnRK2 pathway. 

In response to various stimuli, not only the overall level of PA changes but also different PA species accumulate in the plant cell, providing distinctive regulation of a given process by the interacting specifically with their protein targets. According to Yu et al. [17], in *Arabidopsis*, during the response to salinity, two species of PA are produced preferentially: (16:0/18:2) and (16:0/18:3); their synthesis is catalyzed mainly by PLDα1. Those authors analyzed the profile of PAs in *Arabidopsis* leaves before salt addition and at two time points of the treatment, 30 min and 3 h, and they studied the binding of selected PA species to the mitogen-activated protein kinase MPK6. Recently, Kim et al. [93] showed that PA (16:0/16:0 and 16:0/18:1) is involved in circadian clock regulation in *Arabidopsis*. They found that these two PA species preferentially interact with and affect the function of the core clock regulators LATE ELONGATED HYPOCOTYL (LHY) and CIRCADIAN CLOCK ASSOCIATED 1 (CCA1), both in vitro and in vivo. The PAs inhibited the binding of LHY and CCA1 to the promoter of the *TIMING OF CAB EXPRESSION 1* gene.

In order to reveal which PA species bind SnRK2s and their cellular partners and possibly modulate their signaling, we analyzed the interactions of various PA species with kinases belonging to different groups of the SnRK2 family, the phosphatases ABI1 and PP2CA, AtSCS-A (another inhibitor of SnRK2), and two dehydrins ERD10 and ERD14. The ABA-non-activated SnRK2s, phosphatases, and dehydrins preferentially bound to PA 16:0/18:1. SnRK2.8 showed very weak binding to the some PA species in the dot-blot assay, but the binding to PA-containing liposomes was questionable, which calls into question its biological relevance. SnRK2.6 and AtSCS-A did not interact with PA, nor with any other phospholipid tested. 

We found that PA 16:0/18:1 inhibits the activity of ABI1 and PP2CA and causes translocation of both phosphatases. Moreover, we observed a PA-dependent translocation toward the cell membrane of AtSCS-A. The mechanism of the AtSCS-A translocation seems to be different from that responsible for the PA-dependent translocation of PP2Cs since, unlike the PP2Cs, AtSCS-A does not interact directly with PA or any other lipid studied. Our results showed that PA 16:0/18:1 not only influences the activity and/or localization of the negative regulators of SnRK2s, but also greatly affects the phosphorylation of selected SnRK2 substrates; PA 16:0/18:1 inhibited the phosphorylation of AtSCS-A and two acidic dehydrins ERD10 and ERD14. Moreover, we showed that the phosphorylation of ERD14 affects its binding to PA. Earlier studies [37] showed that, upon salt stress, ERD14 became partially localized to intracellular membranes, possibly endosomes or vacuolar membranes, forming structures resembling the “bulbs” described by References [94,95], as well as next to cellular membranes. A phosphomimetic variant of ERD14 with the S79E substitution showed a much weaker tendency for membrane association, which could be explained by its lower affinity for PA found here. This explanation was further supported by the behavior of ERD14 and ERD14 S79E in protoplasts exposed to PA. While the wild-type dehydrin clustered next to the cellular membranes upon PA treatment, its phosphomimetic variant only showed a weak tendency for such regrouping. These results strongly suggest that the ERD14 phosphorylation at S79 is subject to regulation by PA and, on the other hand, affects the PA-dependent membrane association of the dehydrin. PA is long known to modulate the signaling in an ABA-dependent manner. Here, we showed that it can also fine-tune the signaling executed through the ABA-non-responsive SnRK2s by acting on the kinases themselves, as well as their selected substrates and regulators.

## 4. Material and Methods

### 4.1. Chemicals

The following lipids were used:

PA 18:1 (1,2-dioleoyl-*sn*-glycero-3-phosphate, sodium salt);
PA 16:0/18:1 (1-palmitoyl-2-oleoyl-*sn*-glycero-3-phosphate, sodium salt);
PA 18:2 (1,2-dilinoleoyl-*sn*-glycero-3-phosphate, sodium salt);
PA 16:0/18:2 (1-palmitoyl-2-linoleoyl-*sn*-glycero-3-phosphate, monosodium salt);
PI(4)P (*L*-*α*-phosphatidylinositol-4-phosphate, monosodium salt;
PS 18:1 (1,2-dioleoyl-*sn*-glycero-3-phospho-*L*-serine, sodium salt);
PC 18:1 (1,2-dioleoyl-*sn*-glycero-3-phosphocholine).
PI(4)P was from porcine brain, while the other lipids were synthetic. All of them were supplied by Avanti Polar Lipids, Alabaster, AL, USA. They were dissolved (at 10–50 mg/mL) in chloroform or, in the case of PI(4)P), in chloroform/methanol/H_2_O (20:10:1), sealed tightly in glass test tubes, and stored at −20 °C.

The ProFluor^®^ Ser/Thr PPase Assay from Promega (Madison, WI, USA) was used for the phosphatase activity assay.

cOmplete™ Protease Inhibitor Cocktail and substrates for alkaline-phosphatase (BCIP/NBT Color Development Substrate) were obtained from Roche Diagnostics GmbH (Mannheim, Germany). MBP, lipid-free bovine serum albumin (BSA), primary monoclonal mouse anti-GST antibodies, anti-mouse alkaline phosphatase-conjugated secondary antibodies, and biotinylated thrombin protease were obtained from Sigma-Aldrich or Merck KGaA (Darmstadt, Germany). 

Glutathione-SepharoseTM 4B beads were purchased from GE Healthcare Bio-Sciences AB (Uppsala, Sweden). 

[γ^32^P]ATP was obtained from Hartmann Analytic GmbH (Braunschweig, Germany). 

Chloroform, methanol, HCl, Tris, KCl, MgCl_2_, NaCl, Coomassie Brilliant Blue R-250, mannitol, 2-(N-morpholino)ethanesulfonic acid (MES), Tween-20, and other basic chemicals were obtained from BioShop (Burlington, ON, Canada).

### 4.2. Plant Material

An *Arabidopsis thaliana* T87 suspension culture was used for the isolation of protoplasts. The cells were cultured as described by Yamada et al. [96].

### 4.3. Phospholipid Blot Assay

The dot-blot assay was performed according to Munnik and Wierzchowiecka [97]. A nitrocellulose membrane with immobilized phospholipids (0.25, 0.5, 1.0 μg) was incubated in Tris-Buffered Saline with Tween® 20 Detergent (TBST) blocking solution (20 mM Tris-HCl, pH 7.5; 100 mM NaCl; 0.05% Tween-20; additionally containing 1× x cOmplete™ Protease Inhibitor Cocktail (Roche) and 3% lipid-free BSA) for 5 h at 4 °C and then incubated with respective purified GST-fusion protein (10 μg/mL) in TBST at 4 °C overnight. After extensive washing with TBST (three times for 5 min), the membrane was incubated for 2 h at room temperature with primary monoclonal mouse anti-GST antibodies (Sigma-Aldrich) diluted 1:6000 in TBST supplemented with 1% lipid-free BSA (Sigma-Aldrich). Excess of antibodies was washed out with TBST as described above, and the membrane was incubated with anti-mouse alkaline phosphatase-conjugated secondary antibodies diluted 1:30,000 in TBST buffer for 1 h at room temperature, then washed extensively in TBST (5× 5 min). The secondary antibodies bound to the blots were visualized using substrates for alkaline-phosphatase (BCIP/NBT Color Development Substrate) dissolved in reaction buffer (100 mM Tris-HCl, pH 9.5; 100 mM NaCl; 5 mM MgCl_2_).

### 4.4. Liposome Binding Assay

Liposome binding assays were performed as described before [98,99] with some modifications. Per sample, 640 or 64 nmol of total lipids was used. Phosphatidylcholine (PC), phosphatidylserine (PS) and two PA acyl species (16:0/18:1 and 18:1) were used. Liposomes were composed of PC/PS/PA 16:0/18:1 (4:1:4.4) or PC/PS (4:1). Liposome preparation was carried out at a temperature above lipid transition temperature. Lipids dissolved in chloroform were mixed, dried, and rehydrated in extrusion buffer (25 mM Tris-HCl, pH 7.5; 1 mM DTT; 250 mM raffinose) for 1 h. Unilamellar vesicles were produced using a lipid extruder (with 0.2-mm filters; Avanti Polar Lipids) according to the manufacturer’s instructions. After extrusion, the liposomes were diluted in three volumes of binding buffer [125 mM KCl; 25 mM Tris-HCl, pH 7.5; 1 mM dithiothreitol (DTT); 0.5 mM ethylenediaminetetraacetic acid (EDTA)] and pelleted by centrifugation at 50,000 × *g* for 15 min. Then, they were re-suspended in binding buffer, added to 1.3 μg of purified GST-tagged proteins (NtOSAK, SnRK2.4, SnRK2.6 or SnRK2.8) or GST as a control, and incubated for 45–60 min in a total volume of 50 μL at room temperature. The liposomes were harvested by centrifugation at 16,000× *g* for 30 min and washed once with binding buffer. After addition of Laemmli sample buffer and boiling for 2 min, samples were run on SDS–PAGE, and the gel was stained with Coomassie Brilliant Blue. 

### 4.5. Expression and Purification of Recombinant Proteins

Expression and purification of recombinant SnRK2s was performed according to the procedures described previously by Burza et al. [83] and Bucholc et al. [72], whereas that for recombinant ABI1 and PP2CA was performed according to Krzywińska et al. [69] and [100]. The recombinant ERD10, ERD14, and ERD 14S79E were obtained according to Maszkowska et al. [37]. All these proteins were produced in fusion with GST. Recombinant PP2Cs, ERD10, ERD14, and AtSCS-A were produced in *Escherichia coli* strain Rosetta (Novagen) at 37 °C for 2 h, whereas SnRK2s were produced in BL21 (DE3) strain overnight at 18 °C. The bacterial pellet was resuspended in TBS buffer (20 mM Tris-HCl, pH 8.0; 150 mM NaCl; 1 mM DTT) and incubated with lysozyme (0.1 mg/mL) for 20 min on ice. Next, the suspension was sonicated four times for 1 min each with a Sonifier 250 sonicator (Branson Ultrasonics Corp., Danburg, CT, USA) and centrifuged (12,000 rpm, 15 min, 4 °C). 

All recombinant proteins were purified on Glutathione-Sepharose beads. In the case of AtSCS-A and SnRK2.4 for its structure studies, the GST-tag was cleaved using biotinylated thrombin protease (Sigma-Aldrich) and the enzyme was removed from the cleavage reaction using immobilized streptavidin.

### 4.6. Protein Kinase Assay

The protein kinase assay was performed as described previously [72], with minor changes. Approximately 1–2 µg of a recombinant kinase was incubated with 5 μg of MBP or another protein substrate described in Section 2, 50 μM ATP supplemented with 1 µCi of [γ^32^P]ATP (HARTMANN Analytic) in the absence or presence of a synthetic lipid in kinase buffer (25 mM Tris-HCl, pH 7.5; 5 mM EGTA; 1 mM DTT; 30 mM MgCl_2_). The final volume of the reaction mixture was 25 μL. After 30 min of incubation at 30 °C, the reaction was stopped by addition of Laemmli sample buffer and boiling for 5 min. Proteins were separated by SDS-PAGE and phosphorylated substrate was visualized by autoradiography.

### 4.7. Phosphatase Activity Assay

The activity of recombinant phosphatases was determined in the presence and absence of phospholipid studied using the ProFluor^®^ Ser/Thr PPase Assay (Promega). In the assay, phosphorylated bisamide rhodamine 110 peptide substrate (S/T PPase R110 substrate) or control AMC substrate was used. Dephosphorylation of R110 substrate releases highly fluorescent rhodamine 110, whose fluorescence is directly correlated with phosphatase activity. Briefly, in each reaction 125 ng of recombinant phosphatase was incubated with amounts of lipids as described in Section 2, in 40 mM Tris-HCl, pH 7.5 buffer, supplemented with 20 mM MgCl_2_, 5 µM of R110 substrate, and 5 µM of control AMC substrate, in a final volume of 20 µL, for 30 min at 30 °C. The reaction was terminated by the addition of 10 µL of protease solution and, after 90 min of incubation at room temperature, 10 µL of stabilizer solution was added to the sample. Fluorescence was read using a Beckman Coulter PARADIGM plate reader, at 485/530 nm for the R110 substrate and 360/460 nm for the control AMC peptide.

### 4.8. Circular Dichroism Measurements

Circular dichroism (CD) was determined at 20 °C on a Jasco J-815 CD-spectrometer. Protein solutions (ca. 1 μM) were prepared in 5 mM Tris buffer, pH 7.4, with 100 mM NaCl. Spectra were collected twice with an average time of 2 s for each point and a step size of 1 nm from 195 to 270 nm, before averaging. All spectra were corrected against the buffer. The data were converted to molar residue ellipticity using the relationship [Θ] = θ/(10 × n × l × c), where [Θ] is molar residue ellipticity in (degree·cm^2^·dmol^−1^), θ is the observed ellipticity in millidegrees, n is the number of amino acid residues in the protein, l is the path length in cm, and c is the protein concentration in M. Secondary structure content of the proteins was estimated using the CDNN program (CD spectroscopy deconvolution software).

### 4.9. Transient Expression in Protoplasts

For the analysis of subcellular localization, the protein of interest was expressed in protoplasts transformed with pSITE-2CA vector with complementary DNA (cDNA) encoding ERD14 (described in Reference [37]) or pSAT6-EGFP-N or pSAT6-EGFP-C1 with cDNA encoding AtSCS-A, ABI1, or PP2CA. The protoplasts were isolated from the T87 *Arabidopsis* cells cultured as described by Yamada et al. [96]. They were transformed with the appropriate plasmid according to He et al. [101], with minor modifications. In each transformation, approximately 5 × 10^5^ protoplasts were transfected with 40 μg of plasmid DNA. After transformation, the protoplasts were suspended in Washing and Incubation (WI) solution (0.5 M mannitol; 4 mM MES, pH 5.7; 20 mM KCl) and incubated at 21 °C in the dark for approximately 16 h. Next, the protoplasts were treated or not with PA, as indicated in Section 2, and analyzed using confocal microscopy.

### 4.10. Confocal Laser Scanning Microscopy

The subcellular localization of fluorescent fusion proteins was evaluated using a Nikon C1 confocal system built on a TE2000E platform and equipped with a 60× Plan-Apochromat oil immersion objective (Nikon Instruments B.V. Europe, Amsterdam, The Netherlands). The EGFP fusion proteins were excited with a Sapphire 488-nm laser (Coherent, Santa Clara, CA, USA) and observed using a 515/530-nm emission filter. Confocal images were processed and analyzed using EZ-C1 3.60 Nikon FreeViewer software.

## 5. Conclusions

Phosphatidic acid (PA) 16:0/18:1 interacts with ABA-non-activated SnRK2s and their partners (substrates and regulators), thereby influencing SnRK2 signaling. PA affects phosphorylation and subcellular localization of at least some cellular targets of the ABA-non-activated SnRK2s, as well as the activity and/or subcellular localization of SnRK2 regulators. By affecting both the ABA-non-activated SnRK2s and their substrates and regulators, PA can modulate plant signaling in a complex manner.

## Supplementary Materials

The following are available online at https://www.mdpi.com/1422-0067/21/14/4984/s1: Figure S1. Binding of selected SnRK2s and phosphatases ABI1 and PP2CA to PA 16:0/18:1 monitored by liposome assay using liposomes containing 64 nmol of total lipids. Liposomes containing 64 nM of lipids per sample composed of PC/PS/PA 16:0/18:1 (4:1:4.4) or PC/PS (4:1) were incubated with about 1.3 μg of indicated proteins and then pelleted by centrifugation. Proteins in the pellet (P) and supernatant (S) were separated by SDS-PAGE and stained with Coomassie Brilliant Blue; Figure S2. Effect of PA and PC on phosphorylation of AtSCS-A by SnRK2s. AtSCS-A phosphorylation was analyzed by in vitro phosphorylation assay. The phosphorylation catalyzed by GST-fused kinases SnRK2.4, SnRK2.5, SnRK2.10, or SnRK2.8 was performed using AtSCS-A and [γ^32^P]ATP as substrates in the absence (C) or presence of 50 μM PA or 50 μM PC in reaction mixture. Reaction products were separated by SDS-PAGE, and AtSCS-A phosphorylation was determined by autoradiography; Figure S3. AtSCS-A does not bind phospholipids studied. The binding was analyzed using a phospholipid blot assay, as described in Section 4 and legend to Figure 1. GST-AtSCS-A and GST-SnRK2.4 (as a positive control) produced in *E. coli* were used. Supporting Information, Original images of blots and gels.
