# Supplementary material for: Regulation of ABA-Non-Activated SNF1-Related Protein Kinase 2 Signaling Pathways by Phosphatidic Acid
Source: Int J Mol Sci. 2020 Jul 15;21(14):4984. doi: 10.3390/ijms21144984 (PMC7404309; doi:10.3390/ijms21144984)

Supplementary Materials

Supplemental Figure S1

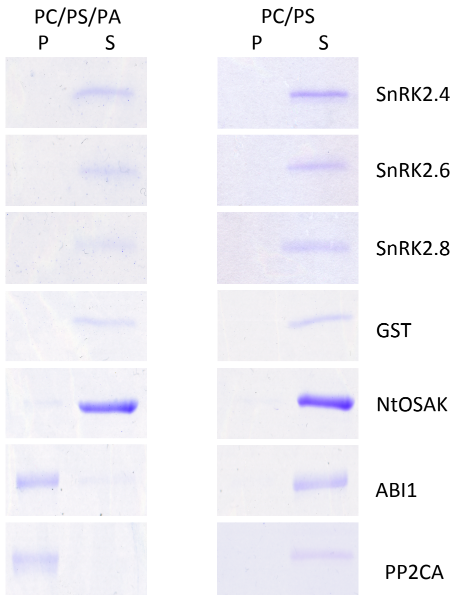

Binding of selected SnRK2s and phosphatases ABI1 and PP2CA to PA 16:0/18:1 monitored by liposome assay using liposomes containing 64 nmol of total lipids.
Liposomes containing 64 nM of lipids per sample composed of PC/PS/PA 16:0/18:1 (4:1:4.4) or PC/PS (4:1) were incubated with about 1.3 µg of indicated proteins and then pelleted by centrifugation. Proteins in the pellet (P) and supernatant (S) were separated by SDS-PAGE and stained with Coomassie Brilliant Blue.

Supplemental Figure S2.

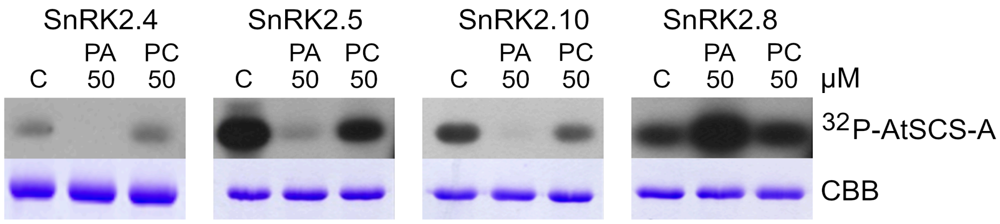

Effect of PA and PC on phosphorylation of AtSCS-A by SnRK2s
AtSCS-A phosphorylation was analyzed by *in vitro* phosphorylation assay. The phosphorylation catalyzed by GST-fused kinases SnRK2.4, SnRK2.5, SnRK2.10 or SnRK2.8 was performed using AtSCS-A and [γ^{32}P]ATP as substrates in the absence (C) or presence of 50 µM PA or 50 µM PC in reaction mixture. Reaction products were separated by SDS-PAGE and AtSCS-A phosphorylation was determined by autoradiography.

Supplemental Figure S2.

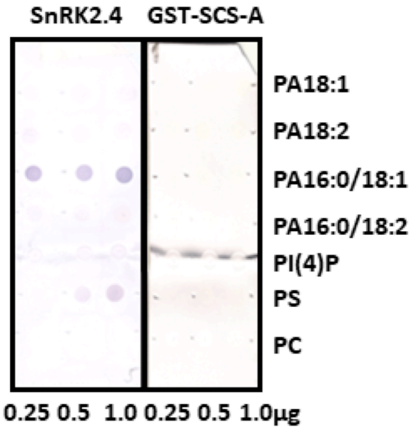

AtSCS-A does not bind phospholipids studied
The binding was analyzed using a phospholipid blot assay, as described in Materials and Methods and legend to Figure 1. GST-AtSCS-A and GST-SnRK2.4 (as a positive control) produced in *E.coli* were used.

Suppplemental Information

Original images of blots and gels

Original data for Figure 1

Fig.1A Phospholipid blot assay

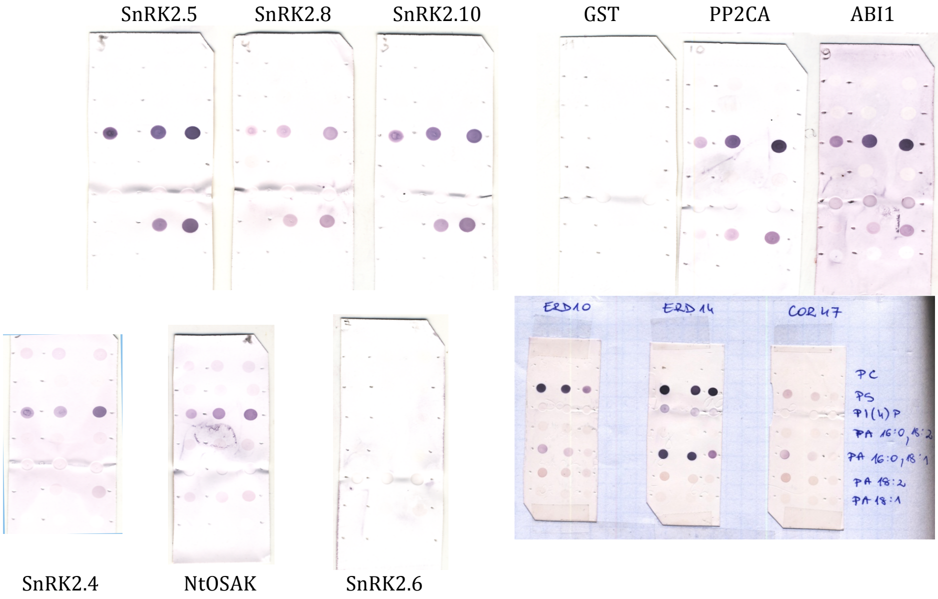

Fig.1B Liposome assay

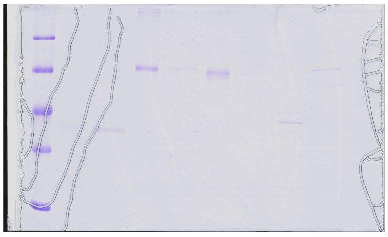

SnRK2.4, ABI1, and PP2CA +PA

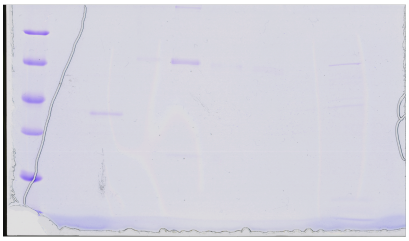

SnRK2.4, ABI1, and PP2CA control

Fig 1C. Liposome assay

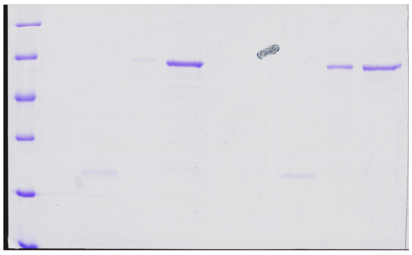

GST and NtOSAK control, and GST and NtOSAK + PA

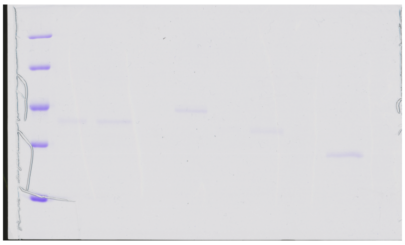

SnRK2.4, SnRK2.6, SnRK2.8, and GST + PA

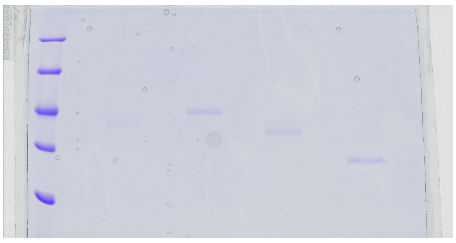

SnRK2.4, SnRK2.6, SnRK2.8, and GST control

Original data for Figure 3

Effect of PA on MBP phosphorylation by SnRK2s

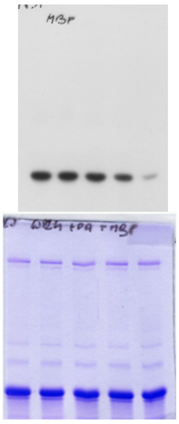

SnRK2.4

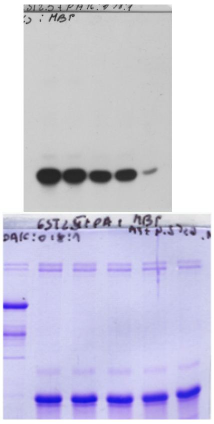

SnRK2.5

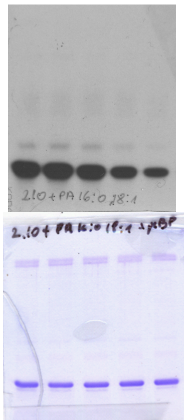

SnRK2.10

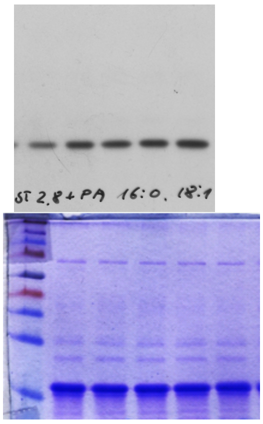

SnRK2.8

Original data for Figure 4

Figure 4A Effect of PA on phosphorylation of ERD10 and ERD14 by SnRK2s

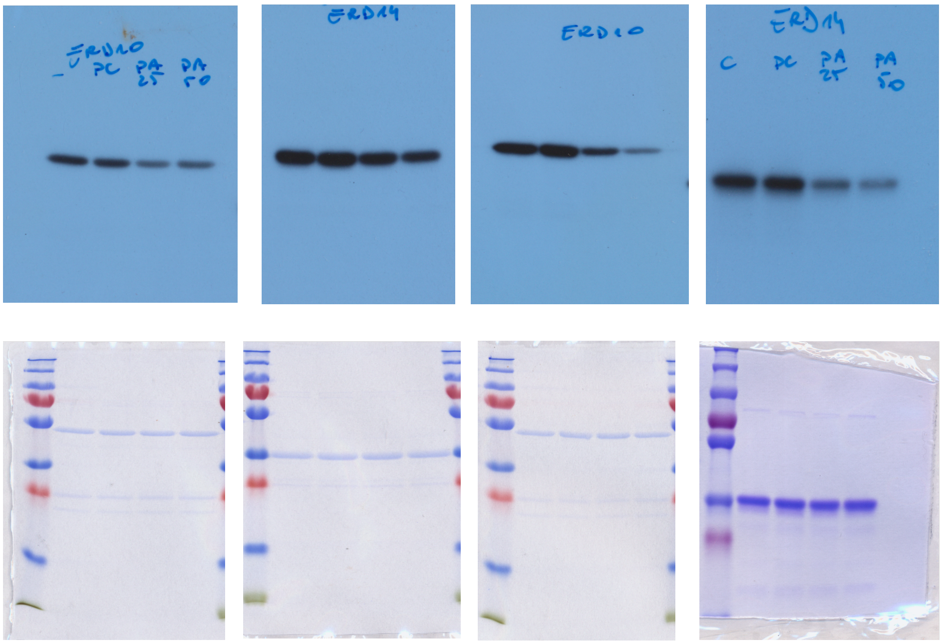

Phosphorylation by SnRK2.4

Phosphorylation by SnRK2.10

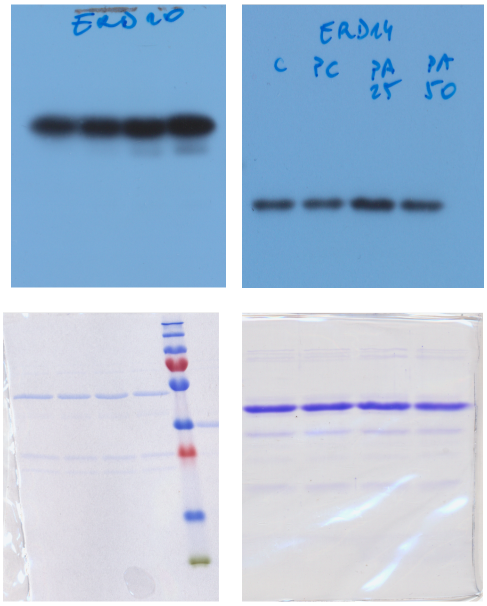

Phosphorylation by SnRK2.8

Figure 4 B Phospholipid blot assay (ERD14 and ERD14 S79E)

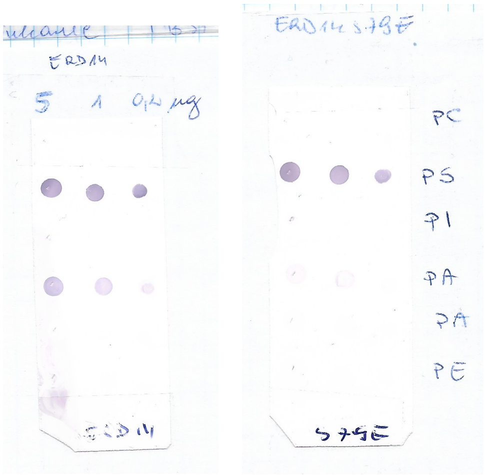

Supplement: Supplementary file 1 [file ijms-21-04984-s001.pdf]
